# An Effective Strategy for the Design of Proteins with Enhanced Mechanical Stability**

**DOI:** 10.1002/anie.200801761

**Published:** 2008-07-29

**Authors:** Alessandro Borgia, Annette Steward, Jane Clarke

**Keywords:** Force microscopy, mechanical properties, protein engineering, titin

There is increasing interest in the investigation of so-called mechanical proteins. These are common in specialized tissues involved in contraction, cell adhesion, and detection of mechanical stimuli, such as skeletal and cardiac striated muscle, connective tissue, and various kinds of epithelia. The development of methods for manipulating single molecules, particularly atomic force microscopy (AFM), allowed the study of the mechanical properties of such proteins.^1^, ^2^ From the response of a protein molecule to an applied external force, it is now possible to determine characteristics such as stiffness and resilience, and, by means of protein engineering methods, it is becoming increasingly clear how these properties depend on the polypeptide architecture, and therefore, ultimately, on the protein sequence.^3^, ^4^ The understanding of protein energetics and folding kinetics is advancing due to an effective combination of experimental and computer-aided theoretical studies, but the rational engineering of protein thermodynamic stability and/or folding mechanisms is still a hard goal to achieve. Moreover, designing novel proteins or even specific properties remains indisputably difficult, despite a few remarkable successes,^5^, ^6^ essentially because a comprehensive and predictive theory of how a given sequence produces a unique structure is still lacking. In the case of mechanical proteins, the understanding of the relationship between sequence, structure, and mechanical properties is incomplete. To our knowledge there are only two examples of proteins which have been specifically and successfully engineered to improve their resistance to forced unfolding.^7^, ^8^ A recent effort used a recombination method, essentially swapping β strands, in an attempt to re-engineer the mechanical properties of two immunoglobulin (Ig) domains from human titin: I27, the paradigm for all protein forced unfolding studies, and a stronger homologue, I32.^9^ Although the engineered proteins had different mechanical strengths to the parent proteins, this methodology had two shortcomings: first, the resultant mechanical stability of the new proteins could not be predicted, and second, none of the engineered changes produced an I27 domain which was more resistant to forced unfolding than the parent protein. Herein we describe a different strategy to achieve this specific aim, namely, to produce a stronger form of I27.

In our successful attempt to re-engineer a fibronectin type III domain (FNfn10, the tenth fnIII domain of human fibronectin) we used previous protein engineering and computational investigation of a homologous domain TNfn3 (the third fnIII domain from human tenascin) to inform our design. TNfn3 displays a mechanical strength significantly greater than FNfn10. We had shown that rearrangement of the core of TNfn3 was the rate-limiting step in the forced unfolding.^10^ Thus, by inserting the core of TNfn3 into the FNfn10 domain, we were able to produce an active, functional FNfn10 domain which had increased mechanical stability: the same as that of TNfn3.^7^ We decided to use the same strategy to re-engineer I27, that is, to replace the load-bearing region of I27 by that of I32, hopefully to produce an I27 with the same load-bearing capacity of I32.

It has been commonly supposed that the A′ and G strands of titin I band domains are the sole load bearing region of the protein.^11^–^13^ Indeed this was apparently the rationale for the strand-swapping procedure of the previous study.^9^ However, by combining protein engineering (phi-value analysis)^14^ and computer simulations, we have previously mapped the positions of I27 that are important for the stabilization of the transition state of the forced unfolding pathway.^15^ It is not only the A′ and G strands that are critical, but also residues in the elements of secondary structure that pack onto these two strands, in particular the E–F loop and the A′–B turn. I32, like I27, is a member of a group of Ig-like domains clustered in the I band of human cardiac titin, and is the strongest of the titin domains characterized to date.^16^ It shares 42 % sequence identity with I27; thus, although the structure of I32 has not yet been resolved, we assumed that the topology of the two proteins and the general features of their transition states were very similar. Based on such characterization and our hypothesis, we replaced the relevant residues in I27 by the corresponding residues found in analogous positions in I32. A total of twelve substitutions were made (Figure 1). Grouped according to their position in elements of secondary structure, these are Y9K/G10D (A–A′), E12T/F14T (A′ strand), V15 A (A′–B), H20T/E22D/I23C (B strand), G66E/M67D/T68A (E–F), and K85F (G strand).

**Figure 1:**
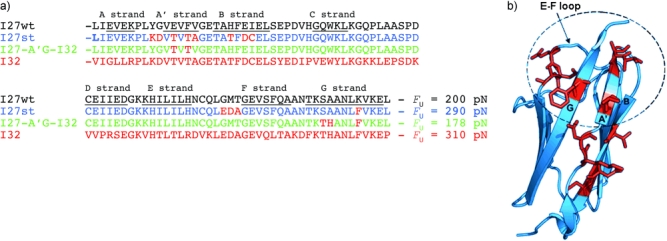
The design of I27st. a) Sequence alignment of I27wt (black), I27st (engineered version of I27, blue), I27-A′G-I32 (previously reported strand-swap mutant,^9^ green), and I32 (red). *F*_U_ is the unfolding force. The twelve substitutions made in I27wt to produce I27st (made on the basis of I32 sequence) are shown in red. b) Positions of substituted residues (shown in red). The circle encloses the C-terminal portion of the protein, in which the key elements for mechanical stability are located: the A′ and G-parallel β strands, the E–F loop, and the A–B turn.

The monomeric mutant protein, optimistically named I27st (st=stronger), could be expressed and purified, and is soluble. The circular dichroism (CD) spectrum showed that the β-sheet structure was similar to that of the parent, wild-type I27 (I27wt), and the fluorescence spectrum for folded I27st was indistinguishable from I27wt (data not shown). Guanidinium chloride (GdmCl)-induced denaturation experiments, monitoring the intrinsic fluorescence of Trp34, showed that the protein is significantly more stable than the parent I27 (Δ*G*_D–N_, 10.0 and 7.4 kcal mol^−1^, respectively; see Supporting Information, Figure S1). The slope of the transition region of the denaturation curve, the *m* value (proportional to the difference in solvent-accessible surface area between the native (N) and denatured (D) states), is the same as I27wt within error. This suggests that the structural features of I27 are unperturbed by the mutations, confirming the results of CD and fluorescence spectroscopy. The increase in stability is due to a significantly slower rate of unfolding. At a concentration of GdmCl corresponding to the midpoint, the reaction is complete after about 10 000 minutes (ca. 7 days), which makes the measurement of the (un)folding rate-constant dependence on denaturant concentration (chevron plot) uncommonly time-consuming for this mutant. (The results of this analysis are summarized in the Supporting Information, Figure S2, together with those of I27wt and I32). I27st displays an unfolding rate extrapolated at 0 m GdmCl (*k*_u_^0^) of 2.6×10^−8^ s^−1^, which is two orders of magnitude slower than I32 (1.9×10^−6^ s^−1^) and four orders of magnitude slower than I27wt (6.7×10^−4^ s^−1^). Surprisingly, whereas the slope of the folding arm (*m*_kf_) of the I27st chevron plot resembles that of I27wt (2.7 m^−1^ vs. 2.5 m^−1^), the extrapolated refolding rate at 0 m GdmCl matches that of I32 (*k*_f_^0^=0.8 s^−1^), despite the fact that 87 % of the residues come from I27 (*k*_f_^0^=250 s^−1^).

Unfolding force was measured using AFM and a polyprotein containing eight repeats of I27st (Figure 2 a; see the Experimental Section) at ten different constant pulling speeds, from 50 to 5000 nm s^−1^. At least 60 peaks, chosen according to standard criteria^17^, ^18^, were analyzed for each pulling speed and used to build unfolding force histograms (Figure 2 b). The modal values of the distributions of unfolding forces for each pulling speed were plotted as a function of the pulling speed to give a force spectrum (Figure 2 c). A second data set was recorded on a different day at six pulling speeds between 100 and 5000 nm s^−1^ and analyzed. The resulting dependence of unfolding force on retraction velocity, calculated independently, virtually overlapped that of the previous experiment; therefore the two data sets were fitted together. The slope of the force spectrum allows the determination of *x*_u_, which represents the distance between the native state and the transition state along the reaction coordinate.^19^ The value of *x*_u_ for I27st is similar to that of I27wt (2.9 and 3.3 Å, respectively).

**Figure 2:**
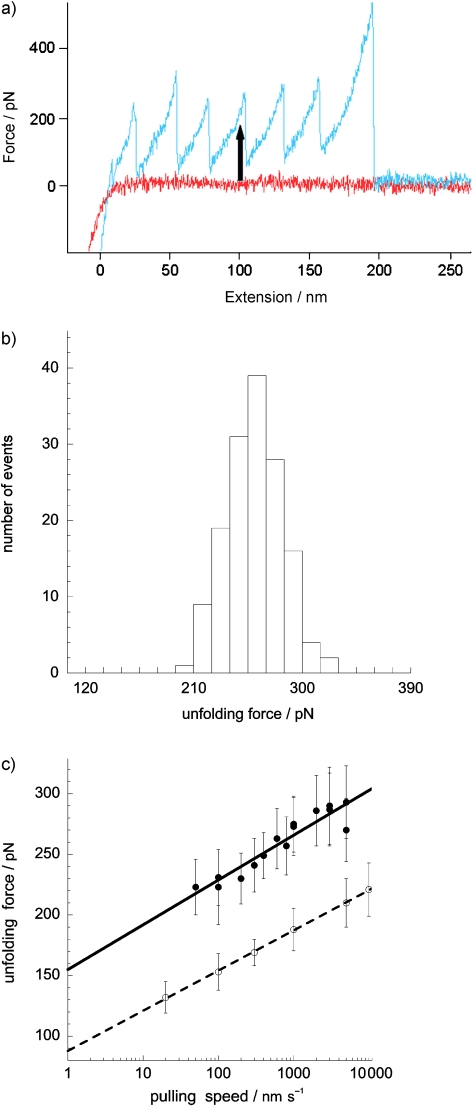
Mechanical properties of I27st. a) Force trace of an I27st eight-mer (blue line) showing the characteristic saw-tooth pattern generated by the consecutive unfolding of protein domains (pulling speed, 1000 nm s^−1^). The modal unfolding force of I27 is shown (black arrow) at the same retraction speed. The red line is the approach trace. b) Histogram of the unfolding force distribution for I27st. 150 unfolding events, with a constant pulling speed of 1000 nm s^−1^; the modal value at this speed is approximately 275 pN. c) Dependence of the modal unfolding force on the pulling speed for I27st (•,—). The same analysis for I27wt (○,– – –, data taken from Ref. 20) is plotted on the same graph, showing that I27st unfolds at a higher force than I27wt at all experimental pulling speeds. The slope of the plot of I27st is very similar to that of I27wt, indicating that the position of the transition state for the unfolding reaction, relative to the native state, is virtually unchanged.

As determined from the force spectra, at all experimental pulling speeds, I27st unfolds at significantly higher forces than I27. How does the unfolding force compare with I32? In our study, at a retraction speed of 2500 nm s^−1^, I27wt unfolds at approximately 200 pN, whereas I27st unfolds at approximately 290 pN. In a previous study of the unfolding of titin domains by Fernandez and co-workers, the unfolding of different titin domains was compared.^16^ According to their data, although the retraction speed was not given, we estimate that, whereas I27wt unfolds at about 200 pN, I32 unfolds at about 300 pN. Thus, with only twelve substitutions, I27 has attained the mechanical stability of I32.

It is interesting to ask why the recent analogous attempt by Li and co-workers to improve the mechanical strength of the same protein was not successful.^9^ The strategy used by Sharma et al. was to replace the whole A′ and G strand of I27 with the corresponding two strands of I32, producing a new sequence (I27-A′G-I32) bearing five mutations, two in the A′ strand (E12T/F14T) and three in the G strand (S79T/A80H/K84F). This work was, in effect, working on the assumption that “the A′–G patch is the key element that imparts the mechanical resistance to I27.”^9^, ^11^–^13^, ^21^ The hybrid protein which had the A′ and G strands of I32 grafted onto I27 did not increase the mechanical strength significantly (unfolding forces of (204±26) pN for wild-type and (178±44) pN for the hybrid protein compared to values of (200±21) pN for I27wt and (290±30) pN for I27st). The previous study did not consider evidence from protein engineering analysis, which showed that side chain interactions between the strands and between the A′ and G strands with the associated E–F and A–B loops also play crucial roles in determining mechanical strength, as reported by Best et al.^15^ Thus, contrary to what was done for I27–A′G–I32 hybrid protein, we limited our mutagenesis of the G strand to its C-terminal portion, which is known to be part of the mechanical clamp, leaving the residues of the N-terminal end unchanged to avoid the risk of compromising the hydrophobic packing with the F strand and the steric features of this region. At the same time, we extended our mutagenesis to one residue beyond the boundary of the A′ strand (V15A, in the A′–B loop) and to the three residues of the E–F loop which are in close proximity to the C-terminal residues of the G strand. We also mutated two residues N-terminal to the A′ strand and three of the N-terminal end of the B strand, which are close to the previous two, to preserve side-chain–side-chain interactions and the steric layout of this region of the protein. The design of our mutant was based on careful evaluation of the different factors we knew could affect the mechanical stability of the engineered protein, taking into account all the background information available about I27wt.

The example of rational protein engineering presented herein indicates that to successfully alter the mechanical properties of a protein, it is vital to have a detailed understanding of the structural features of the transition state structure and a careful evaluation of all the factors which can affect its stability; to this end, a synergy between mechanical *φ*-value analysis and molecular dynamics simulation proved to be a very powerful approach.

## Experimental Section

I27st was produced in three rounds of mutagenesis, performed using the Quick Change kit from Stratagene on the parent plasmid pTII27; the identity of the mutants was confirmed by DNA sequencing, and mutant proteins were produced and purified as I27wt.^22^ The method of construction, production and purification of I27st eight-mer was performed as has been described previously for I27wt.^23^

Denaturant (GdmCl)-induced equilibrium and kinetic (un)folding experiments of single titin domains were determined using a Cary Eclipse fluorimeter, with 2 μm protein in phosphate-buffered saline (PBS; 10 mm sodium phosphate (pH 7.4), 137 mm NaCl, 2.7 mm KCl) and 5 mm 1,4-dithiothreitol (DTT) at 25 °C. Thermodynamic and kinetic data were analyzed using the Kaleidagraph software package (Synergy Software, Reading, PA).

Force measurements were made using a Molecular Force Probe (Asylum Research, Santa Barbara, CA) as described previously.^24^ All the experiments were carried out in PBS at ambient temperature (18–20 °C). A range of pulling speeds between 50 and 5000 nm s^−1^ was used. Silicon nitride cantilevers (Thermomicroscopes, Sunnyvale CA) with a nominal spring constant of 0.03 N m^−1^ were used and calibrated using the thermal fluctuation method implemented in the MFP software.^25^ At least 60 peaks were counted at any given pulling speed to determine a mean unfolding force (the criteria for selecting force–extension traces have been described previously).^18^ Two repeat experiments were carried out to reduce the effect of systematic errors in spring-constant calibration. AFM data were analyzed using Igor Pro software package (Wavemetrics Inc., Lake Oswego, OR).
