# Supplementary material for: An Effective Strategy for the Design of Proteins with Enhanced Mechanical Stability
Source: Angew Chem Int Ed Engl. 2008 Jul 29;47(36):6900–3. doi: 10.1002/anie.200801761 (PMC2852638; doi:10.1002/anie.200801761)
Supplement: Supplementary file 1 [file anie0047-6900-SD1.pdf]

Angewandte Chemie
Eine Zeitschrift der Gesellschaft Deutscher Chemiker

Supporting Information

© Wiley-VCH 2008

69451 Weinheim, Germany

**Supplementary Data for:**

# An effective strategy for the design of proteins with enhanced mechanical stability

**Alessandro Borgia, Annette Steward and Jane Clarke**

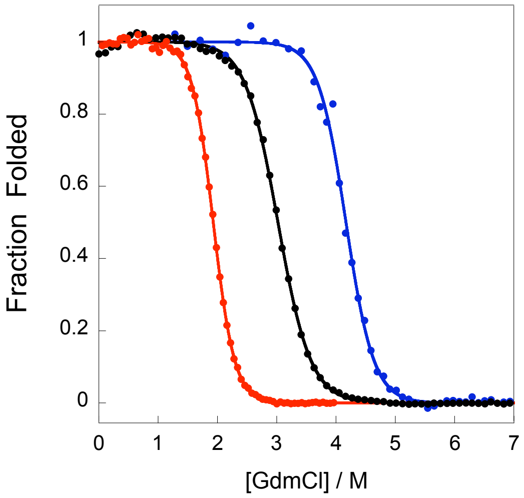


***Supplementary Figure 1*** **Thermodynamic properties of I27st, compared to I27 and I32.** Denaturant-induced equilibrium denaturation experiments of I32 (red), I27wt (black) and I27st (blue). All the three proteins are well described by a 2-state model, indicating that only the native and denatured states are populated at equilibrium. I27st is significantly stabilized compared to I27wt, even though no correlation is expected between thermodynamic and mechanical stability [1] (which depends on the height of the transition state energy barrier).

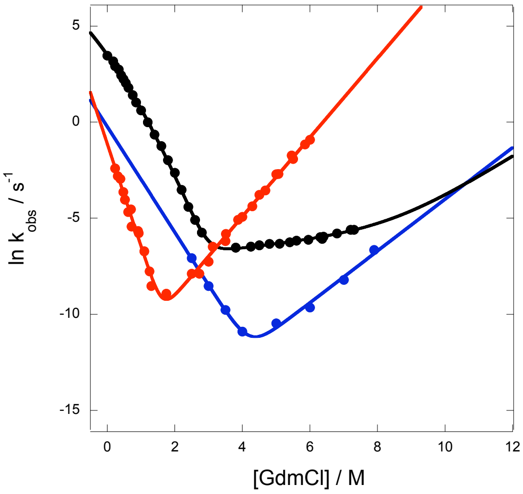


***Supplementary Figure 2*. Kinetic properties of I27st, compared to I27 and I32.** Dependence of the (un)folding rate constants of the three proteins on GdmCl concentration (chevron plots). I27st unfolds more slowly than I27wt; this evidence first suggested that the engineered mutant could be mechanically stronger than the wild-type protein, since a correlation between unfolding rate constants and mechanical stability has been demonstrated in this protein family, because the same regions of the protein unfold in the mechanical and chemical transition state structure for unfolding [1-3].

**References for Supplementary Data**


[1] H. Li, A. F. Oberhauser, S. B. Fowler, J. Clarke, J. M. Fernandez, *Proc. Natl. Acad. Sci. USA* **2000**, *92*, 6527.

[2] H. B. Li, W. A. Linke, A. F. Oberhauser, M. Carrion-Vazquez, J. G. Kerkviliet, H. Lu, P. E. Marszalek, J. M. Fernandez, *Nature* **2002**, 998.

[3] K. A. Scott, A. Steward, S. B. Fowler, J. Clarke, *J. Mol. Biol.* **2002**, *315*, 819.
